# Intertwined Relationship of WWOX and RUNX2 Proteins as a Biomarker for Predicting Response and Survival in Patients With Childhood Bone Cancer in North India: A Pilot Study

**DOI:** 10.7759/cureus.93160

**Published:** 2025-09-24

**Authors:** Priyanka Sharma, Shah Waliullah, Bhavesh Kumar, Ravindra Mohan, Devarshi Rastogi, Dharmendra Kumar, Wahid Ali

**Affiliations:** 1 Pathology, King George's Medical University, Lucknow, IND; 2 Orthopaedic Surgery, King George's Medical University, Lucknow, IND

**Keywords:** ewing sarcoma, north indian population, prognosis, runx2, wwox

## Abstract

Background: Ewing sarcoma (ES) is an aggressive bone cancer that predominantly affects children and adolescents. Despite advancements in treatment, the prognosis for patients with metastatic disease remains poor. This pilot study investigated the expression of WWOX and RUNX2 proteins in ES tissues from patients from North India and their potential as prognostic biomarkers.

Methods: Immunohistochemical analysis was performed on tumor samples from seven ES patients and five age-matched controls.

Results: The results revealed that RUNX2 expression was significantly elevated in ES tissues compared to controls (p = 0.005), while WWOX expression showed no significant difference (p = 0.876). Clinically, 71.4% of patients presented with stage IV disease, and 85.7% had metastasis at diagnosis. The Kaplan-Meier analysis demonstrated poor survival outcomes, with a high mortality rate (85.7%) and reduced median survival time.

Conclusion: These findings suggest that RUNX2 overexpression is a defining characteristic of ES and may contribute to tumor progression and metastasis. In contrast, WWOX does not appear to play a critical role in ES pathogenesis. The advanced stage at presentation and poor survival outcomes underscore the need for earlier detection and biomarker-driven interventions. Further research is warranted to validate RUNX2 as a prognostic biomarker and potential therapeutic target in ES.

## Introduction

Ewing sarcoma (ES) is a translocation-positive small round cell tumor characterized by chimeric fusions between the EWSR1 gene and ETS family members, most notably EWSR1-FLI1, which drives oncogenesis [1]. This disease predominantly affects children and young adults, with a peak incidence during the second decade of life, and typically manifests in bone and soft tissue sites. Clinical outcomes for ES vary significantly; localized disease achieves five-year survival rates of approximately 70%-80%, whereas metastatic cases see survival rates plummet to approximately 20%-30% [2]. As the second most common pediatric primary bone malignancy, ES peaks at approximately 15 years of age and has a global incidence of three cases per million. It accounts for approximately 2% of childhood cancers and primarily affects the trunk and diaphysis of long bones, although 15% of cases involve extraskeletal soft tissues [2]. ES is characterized by aggressive tumor growth and extensive osteolytic bone lesions, leading to pain and fractures. The prognosis is particularly poor when metastatic disease involves the bone or bone marrow, with less than 25% overall survival at five years, compared to approximately 45% for lung metastases [2].

Advances in polychemotherapy, such as the EuroEWING99 protocol using vincristine, ifosfamide, doxorubicin, and etoposide, along with local control measures such as surgery and radiotherapy, have improved survival rates. However, systemic therapy remains crucial because of the systemic nature of the disease [3]. Histologically, ES tumors are composed of small, poorly differentiated round tumor cells that are positive for CD99 staining, aiding diagnosis. The defining molecular event is a specific chromosomal translocation involving the EWS gene on chromosome 22q12 fused with an ETS transcription factor gene, commonly FLI-1, resulting in the EWS-FLI1 fusion protein, which drives tumorigenesis [3]. Bone lesions are dominated by osteolysis due to activated osteoclasts, reinforcing a vicious cycle between tumors and bone-resorbing cells. The tumor microenvironment further supports progression and therapeutic resistance, with hypoxia contributing to chemotherapy resistance, immune escape, and angiogenesis. Investigations into microenvironment-targeting therapies, such as bisphosphonates and RANKL inhibitors, are ongoing [3]. Despite these advances, challenges in treating metastatic disease persist, and outcomes remain poor, underscoring the urgent need for new biomarkers and therapeutic targets.

Against this clinical backdrop, our study specifically aims to explore the roles of WWOX and RUNX2 as potential biomarkers in ES. The WWOX tumor suppressor gene is known to interact with RUNX2, a key osteoblast transcription factor, in osteosarcoma and bone biology [4]; however, its expression and role along the WWOX-RUNX2 axis in ES tissue remains understudied, representing a gap in understanding ES molecular pathology. The rationale for focusing on this axis lies in prior evidence showing that WWOX suppresses RUNX2 activity and thereby reduces metastatic potential in osteosarcoma [4], raising the possibility that a similar mechanism may exist in ES.

At the molecular level, the hallmark EWSR1-FLI1 fusion protein drives tumorigenesis by reprogramming transcriptional programs [5]. Mechanistic studies indicate that EWSR1-FLI1 not only activates oncogenic target genes but also impairs osteoblast differentiation by interacting with regulators such as RUNX2 (Clinical Trial Search: 0d651ebcf2a3, 2.2) [2]. This disruption of normal osteogenic programs by EWSR1-FLI1 provides a compelling rationale for evaluating RUNX2 as both a biomarker and potential therapeutic target in ES [2]. Considering these observations, it is biologically plausible that the fusion not only directly drives malignant transformation but also disrupts osteogenic differentiation by sequestering and inhibiting RUNX2. This disruption may be exacerbated by alterations in WWOX function, potentially worsening dysregulation of osteogenic signaling in ES.

An additional layer of biological complexity involves the tumor suppressor WWOX, which physically associates with RUNX2 and suppresses its transactivation activity. Experimental data from osteoblast and osteosarcoma models have shown that loss or inactivation of WWOX leads to aberrant RUNX2 activity, contributing to oncogenic processes through deregulated osteogenic signaling [4, 6]. Although these findings are robust in osteoblastic systems, the expression and functional interplay of the WWOX-RUNX2 axis in ES tissue have been reported only sporadically and inconsistently, highlighting a significant gap in our understanding. Reports on RUNX2 and WWOX protein expression in ES are sparse and heterogeneous, and inconsistent findings may stem from unique molecular features of ES, differences in tumor microenvironment, methodological variability, or tumor heterogeneity. Consequently, although the inverse correlation between WWOX and RUNX2 is well documented in osteosarcoma, direct evidence of a similar relationship in ES is currently under-characterized and remains to be firmly established.

Taken together, these molecular insights suggest that disruption of the WWOX-RUNX2 axis could play a role in ES biology, but confirmation in well-characterized ES cohorts is needed. This provides a logical bridge to investigating how these molecular alterations relate to clinical behavior and prognosis in our study.

In simpler terms, RUNX2 can be thought of as a “switch” that promotes bone-like, invasive behavior in tumor cells. If this switch remains unchecked, it may drive metastasis and poor survival outcomes. Targeting RUNX2, or restoring its regulation by WWOX, could therefore represent a new therapeutic strategy for ES in the future.

## Materials and methods

Methods

Clinical Information of ES Patients

Seven patients visiting the orthopedic surgery department at King George's Medical University (KGMU), Lucknow, India, between 2019 and 2024, were included after providing informed consent. The sample size was not calculated because this was a pilot study. The inclusion criteria were male and female patients without prior therapy and histologically confirmed ES. Patients with previous treatments, unconfirmed diagnoses, or inadequate documentation were excluded. This study followed the Helsinki Declaration procedures with ethical clearance (approval number: 103^rd^ ECM IIB-Ph. D/P2) from the Institutional Ethics Committee of KGMU for this study. Tumors were classified using the American Joint Committee on Cancer (AJCC) 8^th^ edition staging system for primary bone tumors [7]. The evaluation included patient records, clinical history, examination, and tissue analysis. Immunohistochemistry (IHC) scores were analyzed in a manner blinded to patient identity and clinical outcomes. Patients were monitored for 45 months after the treatment.

Specimen and Tissue Collection

Tissue samples: ES was diagnosed via incisional or trocar biopsy. Formalin-fixed paraffin-embedded (FFPE) samples were used for IHC for protein expression analysis. The control samples were subjected to identical procedures according to the standard protocol.

Immunohistochemical Analysis of WWOX and RUNX2 Proteins in Clinical ES Tissue Samples

WWOX and RUNX2 protein expression in patients with ES was assessed by IHC using FFPE tissue samples. Both the case and control samples were analyzed using anti-human antibodies against the WWOX (Thermo Fisher Scientific Cat# PA5-29701, RRID: AB_2547175 (Waltham, MA)) and RUNX2 (Thermo Fisher Scientific Cat# PA5-82787, RRID: AB_2789943) proteins. Both antibodies were purchased from Thermo Fisher Scientific. The ES tumor grade was determined by histological examination using H&E staining. Two 4 µm sections from paraffin blocks were mounted on charged slides for IHC evaluation. Clinical information was obtained from the pathological records of patients at the Department of Orthopedic Surgery, KGMU. For IHC, the sections were treated with primary antibodies (WWOX and RUNX2) for 90 min at 25°C, processed at 98°C for 15 min, and incubated with a secondary antibody (Thermo Fisher Scientific Cat# 31460, RRID: AB_228341) for 60 minutes, followed by the application of DAB tetrahydrochloride.

Scoring of IHC

A semiquantitative scoring system was used to analyze the intensity of the cells stained with WWOX and RUNX2 antibodies in a tissue section using ImageJ software (National Institutes of Health, Bethesda, MD; RRID: SCR_003070) (20). Histopathological assessments were independently performed in a blinded manner by two senior pathologists. Any discrepancies were resolved through a joint review and consensus to ensure diagnostic reliability. IHC images were analyzed using the ImageJ software. Color deconvolution was applied to separate the DAB chromogen from the hematoxylin counterstain, and the staining intensity and percentage positivity were quantified according to the protocol [8]. Nuclear expression of RUNX2 and cytoplasmic/nuclear expression of WWOX were assessed in representative tumor regions at 20x magnification. Regions of interest (ROIs) were selected by two independent observers, and any discrepancies were resolved by consensus. Standardized thresholds were applied uniformly across all cases.

Statistical analysis

Statistical analyses were performed using IBM SPSS Statistics software, version 27.0; IBM Corp., Armonk, NY; RRID: SCR_002865). The data were classified into quantitative and qualitative variables. Normality was tested using the Shapiro‒Wilk test. Quantitative data are presented as means ± SDs or medians (min-max), and qualitative data are presented as frequencies (%). To compare tissue WWOX and RUNX2 protein expression with that of the controls, we employed Mann-Whitney U tests for continuous variables and chi‒square tests for categorical data. The Kaplan-Meier method was used to estimate the median survival time. Statistical analyses were two-sided, with p<0.05.

## Results

Demographics and clinical characteristics

The study included seven cases of ES and five control subjects. The mean age was comparable between the cases (15.71 ± 7.12 years) and controls (15.60 ± 5.64 years; p = 0.720), indicating that the cohorts were age-matched. The gender distribution was also similar, with males comprising four (57.1%) of the cases and two (82%) of the controls (p = 0.858). Within the case group, the disease burden was substantial. A majority of patients (five, 71.4%) were diagnosed with stage IV disease (TNM IV A/B), while only 28.6% were at stage II. Notably, metastasis was present in six (85.7%) of patients, underscoring the aggressive nature of ES in this cohort. Furthermore, mortality was alarmingly high, with six (85.7%) of patients succumbing to the disease during follow-up. No cases reported a history of smoking, thereby eliminating lifestyle bias. The nearly universal presence of metastasis and high mortality reflects both the delayed diagnosis and the aggressive nature of the disease (Table 1).

**Table 1:** Demographics characteristics of the study population TNM: tumor, nodes, metastasis

Demographic characteristics of the study population			
Demographic variable	Cases	Controls	p-value
Age (years), (mean±SD)	15.71±7.12	15.60±5.64	.720
Gender, N(%)			
Male	4 (57.1)	3 (82)	0.858
Female	3 (42.9)	2 (18)	
Ewing sarcoma patients’ characteristics			
TNM grading			
II A & B	2 (28.6)		
IV A & B	5 (71.4)		
Smoking			
Yes	0 (0)		
No	7 (100)		
Metastasis			
Yes	6 (85.7)		
No	1 (14.3)		
Dead/Alive status			
Dead	6 (85.7)		
Alive	1 (14.3)		

Differential expression of WWOX and RUNX2 proteins in tumor vs. control tissue

**Figure 1:**
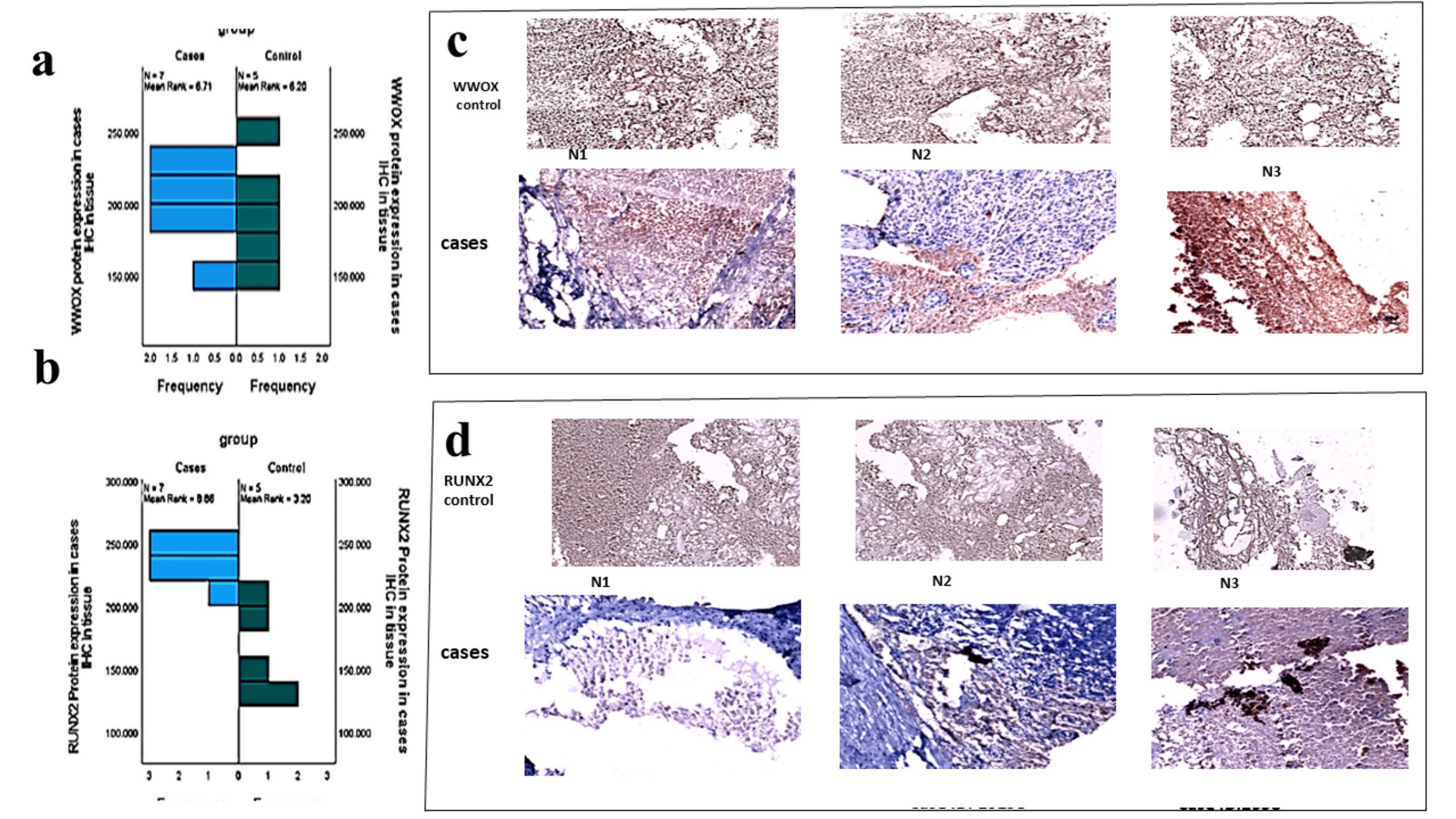
WWOX and RUNX2 protein expression in the tissue a) A graph showing WWOX protein expression in tissue between cases and control; b) A graph showing RUNX2 protein expression in tissue between cases and control; c) Immunohistochemistry (IHC) representative images showing WWOX gene expression between cases and control; d) IHC representative images showing RUNX2 gene expression between cases and control

In Table 2, the median expression levels of WWOX did not exhibit a statistically significant difference between the cases (203.78; range: 151.89-237.61) and the controls (197.02; range: 157.86-241.34; p = 0.876). The graphical representation (Figure 1a) demonstrated substantial overlap, indicating that WWOX is not dysregulated in ES. Representative IHC images corroborated this observation, showing comparable staining intensity in both tumor and normal tissues (Figures 1c, 1d). In contrast, RUNX2 expression was significantly elevated in cases compared to controls (238.28; range: 210.96-249.48 vs. 155.25; range: 122.46-213.42; p = 0.005). The box plot (Figure 1b) illustrated a clear distinction between the groups, and IHC staining revealed pronounced nuclear RUNX2 positivity in tumor cells. These findings suggest that RUNX2, rather than WWOX, plays a pivotal role in the pathobiology of ES. Although WWOX is frequently regarded as a tumor suppressor in other malignancies, its stable expression in this context implies it may not be a critical factor in this tumor type. Conversely, RUNX2 overexpression appears to be a defining characteristic, potentially influencing tumor proliferation, osteogenic signaling, and metastatic potential (Table 2, Figure 1).

**Table 2:** Comparison of WWOX and RUNX2 expression levels between cases and controls The data are expressed as medians with ranges, and p-values are employed to denote the statistical significance of the observed differences between the groups. The analysis involves a comparison of the median (min–max) expression levels of WWOX and RUNX2 in Ewing sarcoma cases (n=7) versus controls (n=5), based on tissue protein levels assessed via immunohistochemistry. Statistical significance is evaluated using the Mann-Whitney U-test, with a threshold of p<0.05 considered indicative of significance.

	Median( min-max)		
Protiein expression	Cases	Control	P-value
WWOX protein expression in tissue	203.78(151.89 – 237.61)	197.02(157.86 – 241.34)	0.876
RUNX2 Protein expression in tissue	238.28(210.96 – 249.48)	155.25(122.46 – 213.42)	0.005

Overall survival in ES patients

**Figure 2:**
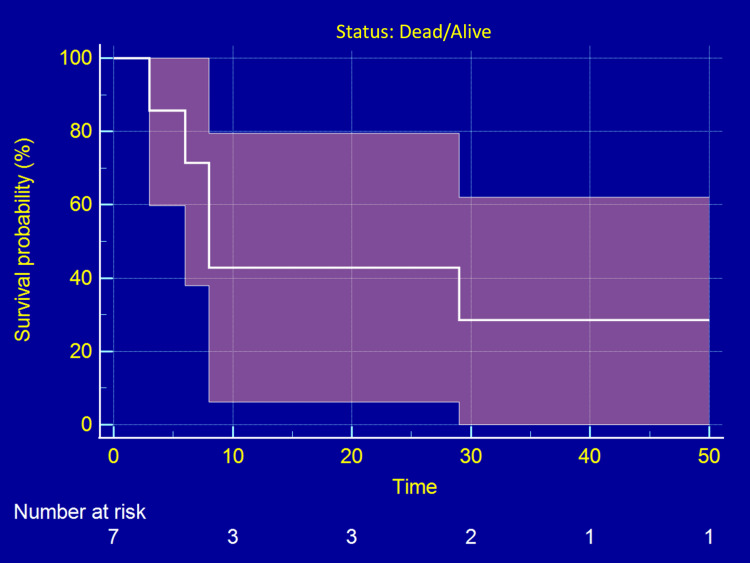
Overall survival (OS) in Ewing sarcoma cases Kaplan-Meier curve showing OS of patients; Time origin = date of confirmed diagnosis; Median OS = 45 months. Numbers at risk are indicated below the x-axis. Patients alive at the last follow-up were censored (marked with tick marks on the curve).

In Figure 2, the Kaplan-Meier analysis demonstrated unfavorable survival outcomes, characterized by a rapid initial decline in survival probability. As evidenced by the six (85.7%) mortality rate presented in Table 1, the majority of patients succumbed to the disease shortly after diagnosis. The median survival time was significantly reduced, reflecting the advanced stage and metastatic nature at the time of diagnosis. The survival curve underscores the bleak prognosis for advanced-stage ES, consistent with global studies that associate metastasis at diagnosis with markedly diminished survival rates (Figure 2).

## Discussion

The current evidence regarding WWOX and RUNX2 in ES is sparse and primarily inferential. ES is an aggressive pediatric bone tumor with limited biomarkers for prognosis and treatment, which underscores the need to explore molecular players with known roles in bone biology. Among these, WWOX and RUNX2 have been repeatedly implicated in other bone-related cancers such as osteosarcoma [6], making them logical candidates for investigation in ES. According to the Open Targets Platform, WWOX shows a modest association with ES, with an association score of approximately 0.037 based on animal model data from the International Mouse Phenotyping Consortium (IMPC) [9]. In contrast, there is no reported evidence or association score for RUNX2 in ES within the platform’s curated datasets, which serves as valuable negative evidence from a therapeutic target prioritization standpoint [3].

In the context of bone tumor research, the interaction between WWOX and RUNX2 has been extensively studied in osteosarcoma. Here, WWOX acts as a tumor suppressor by directly interacting with RUNX2 [6]. Through this interaction, RUNX2 activity is modulated, leading to the downregulation of target genes involved in cell adhesion and motility, thereby suppressing metastatic behavior in osteosarcoma cell lines [6]. Although these findings provide strong mechanistic insights into the tumor-suppressive role of WWOX through modulation of RUNX2, direct investigations of this molecular pathway in ES have not been published. Moreover, other authors further emphasize that while WWOX and RUNX2 are both critical in bone biology and osteosarcoma pathology, where RUNX2 overexpression is typically linked to higher tumor grade, metastasis, and poor prognosis [4, 6, 10], their roles have not been similarly delineated in ES. This suggests that either similar molecular mechanisms are not predominant in ES, or alternatively, they have yet to be thoroughly investigated in this context [11]. The well-documented WWOX-RUNX2 axis in osteosarcoma, demonstrated by WWOX’s ability to suppress RUNX2-driven metastasis, raises the possibility that similar mechanisms might be at play in ES. However, due to the lack of direct studies specifically examining their interaction in ES, any extrapolation remains speculative and warrants further investigation [12, 13]. Taken together, these molecular insights provide the rationale for examining WWOX and RUNX2 in our study and naturally lead to the question of how their expression relates to clinical outcomes in ES [12].

Emerging evidence suggests that RUNX2, a master regulator of osteogenic differentiation, may be enriched in a subpopulation of ES cells that exhibit reduced EWSR1-FLI1 activity, a state associated with increased invasiveness and metastatic propensity [5,14,15]. If validated, RUNX2 enrichment could serve as a predictive marker for metastasis and might indicate a shift toward osteogenic transcriptional programs that favor bone colonization. These insights could ultimately guide the development of new therapeutic strategies targeting RUNX2-mediated pathways to reduce metastatic spread. In simpler terms, RUNX2 may act like a “switch” that pushes tumor cells toward a bone-forming, invasive state, and blocking this switch could potentially slow or prevent tumor spread, offering a future therapeutic opportunity. Mechanistically, RUNX2 may become elevated in ES due to a combination of intrinsic and extrinsic factors. For example, in cells with lower EWSR1-FLI1 expression, the typically repressed osteogenic gene programs become derepressed, allowing for the upregulation of RUNX2 and the activation of osteoblast differentiation pathways [16]. Furthermore, microenvironmental factors such as hypoxia and mechanical cues within bone niches may further stimulate RUNX2 expression, reinforcing an osteogenic and invasive phenotype that promotes metastatic colonization [16, 17]. Taken together, these findings position RUNX2 as both an indicator of underlying cellular plasticity and a potential contributor to the establishment and progression of metastatic lesions.

To ensure cohort homogeneity, diagnostic confirmation typically incorporates established immunohistochemical markers such as NKX2.2 and CD99, along with molecular confirmation of the characteristic EWSR1-ETS fusions [5,18]. This multi-modality diagnostic strategy minimizes misclassification and provides confidence that the observed biomarker associations are specific to bona fide cases of ES. This study aims to offer new insights into the varying protein expression levels of WWOX and RUNX2 in ES, exploring their relationship with clinical outcomes. In particular, the significant increase in RUNX2 protein expression in tumor samples highlights its role as a pro-oncogenic transcription factor in sarcoma development. RUNX2 regulates genes associated with bone formation, invasion, and extracellular matrix remodeling [19]. Consistent with this, its overexpression mirrors findings in osteosarcoma and metastatic carcinomas, where elevated RUNX2 levels are linked to increased aggressiveness and poorer survival rates [14]. The observed association between high RUNX2 protein expression and metastatic presentation in our study further supports its involvement in metastasis.

By contrast, WWOX, a tumor suppressor, showed no significant difference in protein expression between cases and controls in Ewing sarcoma. While WWOX loss is reported in breast, prostate, and lung cancers, its unchanged protein expression [20] here suggests that other pathways may dominate tumorigenesis in ES. This underscores the tumor-type specificity of WWOX function, indicating that therapeutic strategies targeting WWOX may be less relevant in sarcomas compared to epithelial cancers [21]. The clinical implications of poor survival are evident, with a strikingly high proportion of stage IV disease (5/7, 71.4%) and metastasis (6/7, 85.7%) in our cohort, underscoring the need for earlier detection strategies in pediatric and adolescent patients. Indeed, patient stage IV enrichment in study cohorts is closely linked to survival discrepancies. Cohorts with a higher proportion of metastatic patients consistently show worse overall survival compared with those enriched for localized disease, as reflected in multiple survival analyses [22]. Therefore, such enrichment underscores the clinical impact of metastatic dissemination and highlights the urgency for refining risk stratification and therapeutic approaches in ES. In line with this, the Kaplan-Meier survival curve illustrates the rapid progression and poor prognosis, consistent with global survival rates in metastatic ES (~20%-30% at five years). Our findings emphasize the urgent need for biomarker-driven interventions, with RUNX2 potentially serving as a prognostic biomarker and therapeutic target.

A strength of this study is that it is among the first to simultaneously profile WWOX and RUNX2 in Ewing sarcoma, integrating molecular data with survival outcomes. However, an important limitation is the relatively small sample size (n=7 cases), which inherently restricts statistical power and limits the generalizability of the results. Thus, these observations should be regarded as hypothesis-generating. To enhance the reliability and external validity of these findings, future research should incorporate larger cohorts and ideally involve multi-institutional collaborations. In this pilot study, no detectable difference in WWOX expression was observed between cases and controls; however, given the small cohort size, these results should be interpreted cautiously. WWOX is known to suppress RUNX2 transactivation, and this regulatory axis may represent a counter-mechanism that warrants investigation in a larger cohort. Our findings of elevated RUNX2 expression alongside unchanged WWOX levels underscore the need for further studies with adequate statistical power to clarify the role of this pathway in ES.

## Conclusions

This study concludes that RUNX2 is markedly overexpressed in ES tissues and may contribute to tumor advancement, whereas WWOX levels remain stable. Clinically, patients are diagnosed at advanced stages with extensive metastasis and poor survival rates. In summary, this pilot study highlights a significant increase in RUNX2 expression in ES, suggesting its potential role as a prognostic biomarker and therapeutic target. By contrast, WWOX expression did not differ between cases and controls, which may indicate tumor-type-specific biology in ES. Although the small sample size limits the strength of our conclusions, these findings generate important hypotheses regarding the WWOX-RUNX2 regulatory axis and its contribution to disease progression. Future large-scale, multi-institutional studies are warranted to validate RUNX2 as a prognostic marker, clarify the modulatory role of WWOX, and explore whether targeting this pathway can improve clinical outcomes in patients with Ewing sarcoma. These results emphasize the prognostic importance of RUNX2 and the necessity of biomarker-driven approaches to enhance survival in ES.
